# Recharging exhausted parents: How and when involvement in children's education increases working parents' flourishing at home and engagement at work

**DOI:** 10.1002/pchj.753

**Published:** 2024-04-16

**Authors:** Zhuojun Wang, Xinwen Bai

**Affiliations:** ^1^ CAS Key Laboratory of Behavioral Science Institute of Psychology, Chinese Academy of Sciences Beijing China; ^2^ Department of Psychology University of Chinese Academy of Sciences Beijing China

**Keywords:** conservation of resources theory, engagement at work, flourishing, parental burnout, parental involvement in children's education, work–family interface

## Abstract

Parental involvement in children's education is highly valued and encouraged in many societies. While existing research has mainly focused on the positive effects parental involvement has for children, we argue that engaging in such quality parent–child interactions can also be a resource‐gaining process for parents. Drawing on the conservation of resources theory and the work–home resources model, the current study aims to investigate how and when working parents' involvement in children's education enhances their well‐being at home and engagement at work. Using a two‐wave survey of 206 full‐time employees with at least one school‐aged child, our results indicate that for parents experiencing higher levels of parental burnout, involvement in their children's education enhances their flourishing experience at home and subsequently improves work engagement and creative process engagement at work. Overall, our study contributes to the well‐being and work–family interface literature by highlighting the positive effect of parental involvement, an underexplored construct, on working parents' well‐being both at home and in the workplace. This study also provides practical implications for burned‐out working parents that they can benefit from involving themselves in their children's education to cope with and thrive from family demands.

## INTRODUCTION

Regarded as an important part of effective education for children (Hornby & Lafaele, 2011), parental involvement in children's education (PI‐E) has been widely valued and encouraged in many societies. For working parents, home‐based PI‐E has evolved into an integral aspect of parent–child interactions. Many parents devote a substantial portion of their time after work to assisting with their children's homework, reviewing the next day's lessons, or making study plans for their children. Indeed, there is a global trend for parents to become increasingly involved in their children's education at home (Herrold & O'Donnell, 2008; United Nations, 2020; Williams et al., 2002).

In response to this increasing trend, scholars have conducted numerous studies investigating the effect of PI‐E. The majority of research focuses on the relationship between PI‐E and children's academic outcomes. It has been consistently indicated that PI‐E enhances children's academic achievement (see Fan & Chen, 2001; Jeynes, 2007; Pomerantz et al., 2007; and Wilder, 2013, for intensive meta‐analyses), and improves children's well‐being in terms of better mental health (Wang & Sheikh‐Khalil, 2014; Wong et al., 2018), higher self‐esteem (Ho, 2003; Krauss et al., 2020), and better emotional functioning (Barger et al., 2019; Wang & Sheikh‐Khalil, 2014). However, while most studies focus on the positive effect of PI‐E for children, it remains underexplored whether and how parents themselves could benefit from it.

Understanding what effect PI‐E will generate for parents is of significance because PI‐E could be an overlooked resource that is vital to parents' eudaimonic well‐being. Eudaimonic well‐being goes beyond the surface hedonic level of feeling good and is based on the fulfillment of meaningful human needs, such as personal growth and self‐realization (Ryff, 1989). As an essential indicator capturing eudaimonic well‐being (Drake et al., 2021; Liu et al., 2022; Ng et al., 2020; Schotanus‐Dijkstra et al., 2015), flourishing encompasses both intrapersonal aspects (e.g., finding meaning and purpose in life) and interpersonal aspects (e.g., fostering positive relationships and contributing to others' well‐being; Diener et al., 2010). Some studies imply that PI‐E can influence both aspects of flourishing. For example, spending more time with children is associated with parents' experiences of meaning in life (Nelson et al., 2013). Since educating their children is one of the most valuable life goals for most parents (Fave & Massimini, 2004), active involvement in their children's education can help parents better understand their life purpose. PI‐E is also closely related to the social role of parents. Parents believe that their active involvement can help their children succeed in school, thus facilitate parental role construction and increase parental self‐efficacy (Green et al., 2007). While existing studies might provide some indirect evidence implying that PI‐E could contribute to important components of flourishing, to our best knowledge, the direct exploration of how PI‐E contributes to parents' eudaimonic well‐being is lacking.

**FIGURE 1:**
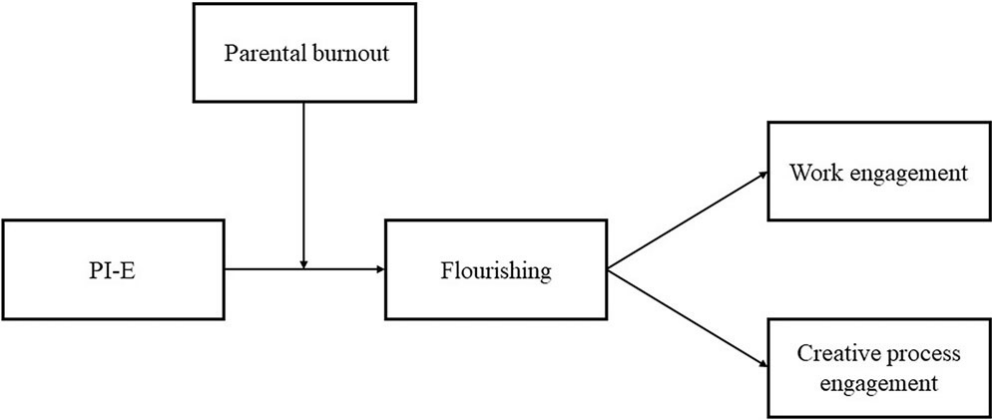
Theoretical model. PI‐E, parental involvement in children's education.

In the current study, we argue and provide evidence that PI‐E facilitates parents' flourishing experience. Drawing on the conservation of resources (COR) theory (Hobfoll, 2001; Hobfoll & Kent, 1989) and the work–home resources (W‐HR) model (ten Brummelhuis & Bakker, 2012), we propose that PI‐E functions as a resource‐gain process that enhances working parents' flourishing experience at home, which in turn improves their engagement at work. Furthermore, we propose that parental burnout—an exhausted state caused by a chronic mismatch between perceived stressors and resources in parenting (Mikolajczak et al., 2021; Mikolajczak & Roskam, 2018)—will act as an important boundary condition in this resource‐gain process. Following the gain paradox principle of COR (Hobfoll et al., 2018), which states that individuals under resource‐loss conditions will weigh higher on resource gain (Hobfoll et al., 2018), we theorize that individuals with a high level of parental burnout can gain more resources and obtain a greater flourishing experience by getting involved in their children's education. Figure 1 depicts our full theoretical model.

### Parental involvement in children's education

PI‐E can be defined as “parents' interaction with schools and with their children to benefit their children's educational success” (Hill et al., 2004, p. 2). Definitions of PI‐E are varied. Grolnick and Slowiaczek (1994) describe PI‐E as parents' commitment of resources, including time, energy, and money, to the academic context of their children's lives. It is necessary to point out that Grolnick and Slowiaczek's (1994) definition is broad and considered to be general and inclusive (Boonk et al., 2018), without delving into the specific and discrete behaviors parents demonstrate in daily life with their children.

Other researchers have taken a different perspective to identify what activities parents are most concerned about when they wish to be involved in their child's learning. Following this approach, scholars have distinguished two forms of PI‐E, that is, school‐based PI‐E and home‐based PI‐E (Hornby & Blackwell, 2018; Hornby & Lafaele, 2011; Pomerantz et al., 2007). School‐based involvement encompasses activities undertaken by parents at school, requiring parents to make connections with schools. This could involve attending a parent–teacher conference, observing the child in class, or participating in school clubs or activities. Home‐based PI‐E, on the other hand, occurs between parents and children and usually takes place at home. The primary goal of parents is to actively engage in their children's learning activities (e.g., discussing academic issues with children, helping with children's homework). Home‐based PI‐E covers a wide range of issues, such as home supervision, emotional engagement regarding school problems, assisting their children with school assignments or homework, and frequent parent–child communication about school (Benner et al., 2016; Gan & Bilige, 2019; Yotyodying & Wild, 2016).

As mentioned earlier, we aim to reveal the positive effect of family involvement on parents' well‐being and explore its potential spillover effect into the workplace. Therefore, we specifically focus on home‐based PI‐E in the present study. By doing so, we can better reveal the impact of concrete family events on parents' well‐being.

### PI‐E and parents' well‐being: A eudaimonic perspective

The association between parenthood and well‐being is a focus point for researchers as well as the general public. In parenting literature, scholars have measured well‐being through indicators such as anxiety, depression symptoms, happiness, satisfaction, and stress (Nelson et al., 2014; Nomaguchi & Milkie, 2020). These measurements align with the definition of hedonic well‐being, emphasizing emotional experiences—enjoying life, maximizing pleasure, and minimizing pain (Diener et al., 1999). Extant studies have suggested the negative relation between PI‐E (e.g., helping with children's homework) and parents' hedonic experiences. For instance, Pomerantz et al. (2005) found that mothers would experience more negative emotions on days when their assistance with children's homework was high. This negative effect may stem from parents' irritation and annoyance when seeing their children's frustration and giving up on completing homework, which may be regarded as signals that children are not working constructively. Similarly, a recent daily diary study suggested that parents were experiencing higher parental negative affect and lower parental positive affect during the days when they had more parental involvement in children's education (Schmidt et al., 2021).

Though the effect of parenting on parents' hedonic well‐being is well‐established, explorations between family events and eudaimonic well‐being are sparse. While parents are likely to experience less hedonic experiences from educating their children at home, they might benefit from such activities in terms of eudaimonic well‐being. Eudaimonic well‐being is conceptualized as feelings of purpose in life and self‐actualization, representing a life lived in accord with one's true self (Brajša‐Žganec et al., 2022; Ryan & Deci, 2001; Ryff, 1989). It is theorized to be positively related to parenting and parenthood because becoming a parent often correlates with increased purpose in life and personal growth, which are considered as important components of flourishing. A longitudinal survey of 50 pregnant couples shows that both fathers and mothers' ratings of eudaimonic well‐being increased after having a child (Brandel et al., 2018). Research indicates that a mother's definitions of happiness incorporate more eudaimonic aspects, such as personal growth, purpose, and mastery, after 6 months of parenthood (Fave et al., 2013). Another study suggests that parents report experiencing more meaning in life during time spent with children (Nelson et al., 2013).

It is understandable that while parenting can be a source of various aspects of eudaimonic well‐being for parents, they may find it challenging to derive meaning in life or achieve personal growth from parenting activities (Morse & Steger, 2019). For instance, as Nomaguchi and Brown (2011) indicate in their study of the relationship between a mother's education level and the outcomes of parenting, mothers with a college degree or higher experienced more parental role captivity and derived less meaning in life from parenting compared to those with lower levels of education. However, this study focuses on routine parenting events or general parent–child interactions. For example, participants (i.e., mothers of young children) were asked to report their experiences of parenting focusing on their concerns with childcare‐related issues, such as children's safety or potential troubles, as well as how rewarding they found general parent–child interactions, such as doing things, sharing interests, or communicating with their children.

In contrast to the routine parenting activities or general parent–child interactions, PI‐E is believed to be a type of quality parental activity that can enhance parents' eudaimonic well‐being. Compared to childcare or simply doing things with children, PI‐E provides parents with more opportunities for meaningful communication, enabling them to understand their children's behavior and address potential challenges in their academic endeavors. As a result, parents can better acknowledge how their children behave at school and whether their children have difficulties in their daily studying. The interactive nature of PI‐E fosters positive parent–child relationships (Larson & Richards, 1994), contributing to the interpersonal aspects of flourishing (e.g., fostering positive relationships and contributing to others' well‐being). Besides, PI‐E is also regarded as a quality parental activity because engaging in children's education is thought to enhance children's human capital (Milkie et al., 2010), which is more closely related to parents' expectations of “being good parents” compared to other routine parenting events.

In the present study, we argue that PI‐E could facilitate parents' flourishing feelings at home by positively impacting the eudaimonic component of flourishing, specifically the meaningfulness of life and role fulfilment (Ryan et al., 2013), for two reasons. First, involvement in children's education helps parents achieve meaningfulness in life. Theoretical and practical research suggested that pursuing life goals helps an individual achieve his or her meaning in life (Bühler et al., 2019; Emmons, 2003; Steger, 2009) and thus benefits employee well‐being (Eldor et al., 2020). Having children provides parents with important goals to pursue (Nelson et al., 2014), and educating their children can be an important goal. Choi et al. (2016) collected data from 603 parents with 2–4 weeks of an experience sampling method. Their result indicated that, while engaging in studying, adults reported a significantly higher level of meaning, particularly when they were interacting with their children. Therefore, via pursuing life goals, parents could derive meaning in life from PI‐E.

Second, PI‐E helps parents fulfill their social role by achieving their parental responsibilities and also meeting their expectations of being good parents, which is crucial for the interpersonal aspect of flourishing. For parents, an important aspect of parental responsibilities is to make sure that their children are doing well in life (Milkie et al., 2010; Steger, 2009). Green et al. (2007) also indicated that “Parental role activity beliefs included parents’ beliefs about what they should do and how active they should be in relation to their children's education” (p. 536). Moreover, academic achievement is undoubtedly one of the most important patterns depicting whether school‐age children are successful at school. Given the important role of PI‐E in children's academic success (Fan & Chen, 2001; Hornby & Blackwell, 2018; Jeynes, 2007; Pomerantz et al., 2007; Wu et al., 2022), involvement in children's education helps parents meet their expectations of being good parents by fulfilling their parental responsibilities of ensuring that their children are doing well at school.

Therefore, we propose the following hypothesis:

Hypothesis 1 Parental involvement in children's education is positively related to flourishing experience at home.

### Moderating role of parental burnout: From the perspective of COR theory

COR theory is one of the most influential theories explaining human stress and well‐being. According to Halbesleben et al. (2014), resources are anything perceived by the individual to help attain his or her goals. The gaining of resources could lead to positive well‐being outcomes in employees' work–life interface (Diener et al., 2010; Fredrickson & Losada, 2005; Wayne et al., 2020). As mentioned earlier, by getting involved in children's learning activities, parents are able to gain valuable psychological (i.e., meaningfulness of life) and social (i.e., fulfillment of social roles) resources, and thus are more likely to experience flourishing.

However, it is worth noting that PI‐E also consumes physical, psychological, and financial resources. Understandably, parents need to invest considerable time and energy to cultivate children's talents and skills (Doepke & Zilibotti, 2019), and the extent to which PI‐E increases the flourishing experience may depend on the balance of the amount of resources gained from and that invested in parental involvement. PI‐E significantly increases the flourishing experience only when the former is greater than the latter. In other words, the resource‐gain effect of PI‐E is not equal for all parents. This speculation is consistent with COR's resource‐gain paradox (Halbesleben et al., 2014), which asserts that people's current resource state would inversely affect how they weigh resource conservation and acquisition. To be more specific, people would weigh higher on resource gain if they are in the state of resource loss. Hobfoll et al. (2018) stated that “the infusion of resources for those with few resources can have a powerful impact in engaging gain momentum and strength” (pp. 105–106), and “the motivation to build a resource gain cycle will increase when losses occur and will have higher payoff under high‐stress conditions” (p. 107). These propositions have been largely supported by extant studies. For example, Lim et al. (2020) intensively review empirical studies published between 1997 and 2019 and conclude that employees who lack information resources in an organization (e.g., facing uncertainty) indeed weigh higher on the resource‐gain process and increase their motivation to actively acquire resources.

Parental burnout occurs when a lasting mismatch exists between perceived stressors and resources in the parenting domain (Mikolajczak et al., 2021; Mikolajczak & Roskam, 2018). Parents with high levels of burnout have two salient symptoms (Kerr et al., 2021; Mikolajczak & Roskam, 2020) that signal that they might be facing a greater loss of resources. The first symptom is emotional detachment from children. For burned‐out parents, the emotional distance from children would drive them either to escape or to detach themselves emotionally from their children. The second symptom of parental burnout is feelings of inadequate parenting and parenting inefficacy (Mikolajczak & Roskam, 2018). Clearly, these symptoms are not conducive to parents maintaining high‐quality relationships with their children (Mikolajczak et al., 2018), which happens to be highly relevant to parents' life goals (Fave & Massimini, 2004). As a result, parental burnout will significantly prevent parents from gaining meaningfulness of life and fulfillment of parental role (Mikolajczak et al., 2021; Roskam et al., 2018), and put them at the disadvantage of chronic and continuing resource losses.

This will give rise to a seemingly paradoxical yet reasonable expectation: highly burned‐out parents are more likely to benefit from actively getting involved in children's learning activities. On the one hand, those with high levels of parental burnout are in greater need of resource gain in terms of meaningfulness of life and fulfillment of parental roles since they are lacking them. On the other hand, as we have argued in deducing Hypothesis 1, PI‐E can enhance the flourishing experience because doing so can help parents obtain life meaningfulness and fulfill their parental role. Taken together, what PI‐E generates is what highly burned‐out parents cherish most. Put differently, they may paradoxically undergo a more salient resource‐gaining process from PI‐E and enjoy a greater flourishing experience.

To summarize, drawing on the gain paradox of COR theory, we expect that burned‐out parents are more likely to experience a salient feeling of meaningfulness and role fulfillment by actively getting involved in their children's education. Given that meaningfulness of life and fulfillment are regarded as important components of the flourishing experience (Ryan et al., 2013), we propose that individuals with higher levels of parental burnout may derive more salient flourishing feelings from PI‐E. Therefore, we hypothesize that:

Hypothesis 2 Parental burnout will moderate the effect of parental involvement in children's education on flourishing experience in that such effect is stronger when parental burnout is high rather than low.

### Spillover effect of PI‐E on work engagement and creative process engagement

As indicated above, working parents could attain the flourishing experience from PI‐E. In this section, we adopt the W‐HR model (ten Brummelhuis & Bakker, 2012) to explain how flourishing conveys the spillover effect of PI‐E at home on workplace engagement amongst working parents.

Extending from the COR theory, the W‐HR model's core premise is that work or home resources will increase personal resources, and those personal resources are subsequently beneficial for improving home or work outcomes (ten Brummelhuis & Bakker, 2012). For example, positive child‐related events can be seen as an important source of family resources because such events can help parents obtain more personal resources, such as positive feelings and energies (Du et al., 2020). In turn, these personal resources enable working parents to function well when they are back to work. In this family‐to‐work spillover process, personal resources serve as an indispensable bridge linking family and work domains (Hobfoll, 2002; ten Brummelhuis & Bakker, 2012). In the present study, we focus on two forms of workplace engagement that are closely related to personal resources: the positive state of work engagement and the positive behavior of creative process engagement (Bakker & Demerouti, 2008). We argue that, as a synthesis of various personal resources, flourishing can also function as a link in the family and work interface of working parents. It can facilitate the transfer of resources from family to work, enhancing their work engagement and creative process engagement.

Regarded as an attitudinal outcome (ten Brummelhuis & Bakker, 2012), work engagement depicts employees' positive, fulfilling work‐related states of vigor, dedication, and absorption (Bakker & Demerouti, 2008). It is a function of being psychologically present at work, which reflects whether individuals are “fully there” in the present moment. As the combination of feeling good and functioning effectively (Diener et al., 2010; Keyes, 2002), the flourishing experience enables people to experience positive affect (Bakker & Oerlemans, 2011). The positive affect generated at home can be carried over to boost work absorption (Rothbard et al., 2021), which is an important dimension of work engagement. Besides, flourishing feelings could also make people feel competent, filled with positive emotions and function well psychologically and socially (Keyes, 2002). Flourishing parents would hold positive feelings of functioning about their family, having satisfying relationships with their family members. These feelings can make them less disturbed or interrupted by family issues, and therefore more engaged at work. Extant study has already indicated that the harmonious feelings that working parents have towards their family are vital and can contribute to the family‐to‐work resource spillover process. Y. Tang et al. (2017) found that employees who are satisfied with their marriage show higher levels of family‐to‐work resource spillover. Lu et al. (2011) found that the feeling of control and mastery in the family area could be treated as an important resource, which is a positive influencing factor on employees' work engagement.

From the perspective of the W‐HR model, work engagement is an attitudinal work outcome that refers to beliefs and feelings valued by employees, other than their actual behavior at work. In addition to routine work issues, creative work is also an important aspect for employees. Employees can have satisfying feelings when they engage in routine work issues and may refuse to spend more time on creative methods or processes. Therefore, work engagement may not capture a more general picture of how employees are feeling and what they are doing at work. To this end, we incorporate creative process engagement into our model. Slightly different from work engagement, creative process engagement represents employees' engagement and effort investment behavior in creativity‐relevant activities, including problem identification, information searching and encoding, and idea generation (Harrison et al., 2022; Zhang & Bartol, 2010). For most employees, it is easier and more comfortable to carry out their work in routine ways rather than trying to come up with novel solutions (Shalley & Gilson, 2004). Therefore, engaging in creative processes requires more investment of significant personal resources and resources‐drained employees are less likely to engage in creative processes at work (Babalola et al., 2021). As indicated above, individuals are likely to experience positive affect when they flourish. Positive emotions affect an individual's cognitive process, which could lead employees to taking a “loose” approach towards creativity (Kaufmann, 2015). While experiencing positive feelings, people are willing to pursue processes with more creativity and novelty (Amabile et al., 2005; Fredrickson, 2001; Rego et al., 2018). Therefore, flourishing employees will be more likely to engage in creative processes.

To summarize, employees who experience flourishing at home own abundant personal resources that they can rely on in the workplace. We propose that these flourishing employees will demonstrate a better state of work engagement in general and a higher level of engagement in creative processes in particular. We hypothesize that:

Hypothesis 3 Flourishing is positively related to work engagement (H3a) and creative process engagement (H3b).

Hypothesis 4 Parental involvement in children's education is indirectly and positively related to work engagement (H4a) or creative process engagement (H4b) through flourishing.

The aforementioned hypotheses propose that PI‐E would enhance parents' work engagement (H3a) and creative process engagement at work (H3b) through flourishing, and that parental burnout moderates the relationship between PI‐E and flourishing (H2). Taken together, the above hypotheses suggest that PI‐E should yield a stronger indirect effect on work engagement or creative process engagement when parental burnout is high rather than low. We, therefore, raise our final hypothesis to examine whether the spillover effect of PI‐E through flourishing on each form of engagement at work varies depending on the level of parental burnout.

Hypothesis 5 Parental burnout moderates the indirect effect of parental involvement in children's education on work engagement (H5a) or creative process engagement (H5b) such that each indirect effect is stronger when parental burnout is high rather than low.

## METHODS

### Sample and procedure

To test our hypotheses, we surveyed child‐bearing employees in China at two different time points with 1‐month interval. We recruited working parents through Credamo (www.credamo.com), a reliable Chinese online data‐collection platform, which is similar to Qualtrics and commonly used to recruit participants in China (Gong et al., 2020; Guo et al., 2022). Participants were presented with and signed the informed consent about the study's purpose and procedure. To participate in our survey, participants were required to be full‐time employees living with at least one child aged 6–10 years. This specific age group was selected according to the definition of a school‐age child in the review of Erickson et al. (2010). In this review, Erickson et al. (2010) divided work–family experience by six life stages with children's age. Compared with parents in the pre‐school child stage (under the age of 6 years), Erickson suggested that parents in the school‐age child stage (over the age of 6 years) would spend more time on their children's extracurricular activities. Thus, the age of 6 years may be a turning point for parents with children, after which they become more involved in their children's education from daily childcare or household tasks and have different work–family experiences. Referring to their definition of the school‐age child stage (youngest dependent child aged 6–12 years), we finally targeted a specific age range of children from first to third grade in primary school (youngest dependent child aged 6–10 years).

At Time 1, we recruited 375 employees online in April 2021. Among them, 62 did not meet our criteria (neither working full‐time nor living with at least one child aged 6–10 years), and 13 failed attention checks, resulting in a valid response rate of 80%. With the remaining 300 participants, we collected their data on PI‐E, positive parent–child interactions, parental burnout level, and demographic characteristics, including gender, age, socioeconomic status (SES) and their children's learning performance.

At Time 2, all 300 respondents who had provided valid responses in the first survey were invited, and 240 of them participated. Four of them failed the attention checks, thus were dropped. In addition to the attention checks, we also asked them to report gender in order to detect unfocused respondents. Of the remaining 236 respondents, 30 responded with inconsistent answers on gender between Time 1 and Time 2, thus were further deleted from the final sample. As a result, a total of 206 respondents gave valid responses in both surveys, returning a valid response rate of 68.67% for our final sample. We collected participants' flourishing feelings at home, whether they are their children's primary carer, work engagement, creative process engagement, job autonomy, and job demands. For the final valid sample (*n* = 206), 37.4% of participants were male, and their mean age was 33.07 years (*SD* = 4.44 years).

We conducted the attrition analysis to examine whether participants in the final sample (*n* = 206) differed from those who dropped out after the first data collection (*n* = 94). Results indicated that there were no significant differences between them in terms of PI‐E (*t* = 0.89, *p* = .37), parental burnout (*t* = 1.03, *p* = .30), or any demographic characteristics (*p*s >.2). Taken together, these results suggested that participants randomly dropped out of the study.

### Measures

#### Parental involvement in education

By definition, home‐based PI‐E consists of interactions that take place between child and parent outside of school and focus on the child's learning‐related activities (Green et al., 2007). Researchers usually measure home‐based PI‐E by assessing how frequently parents are involved in children's learning‐related activities (Yotyodying & Wild, 2016). Given that the content of children's learning‐related activities is culturally dependent (Ip et al., 2018), we measured PI‐E with seven activities that Chinese parents of young children are most concerned about and often engaging in: completing/checking homework, making a study plan, reviewing today's lessons, previewing the next day's lessons, dictating words, word recognition/numeracy, and reciting ancient poems. Participants indicated how often they had been involved in each of these learning activities with their children in the past 3 months on a seven‐point scale (1 = *Never*, 7 = *Almost every day*; α = .85). We averaged all the items, and a higher score indicated higher levels of PI‐E.

To assess the construct validity of our seven‐item scale, we conducted a pilot study by obtaining a non‐overlapping sample consisting of 122 working parents who were living with at least one child aged 6–10 years (Grade 1–3) through the same platform (i.e., Credamo) for our main study. We relied on Ip et al.'s (2018) measure to assess convergent validity because their items were developed to measure Chinese parents' involvement in children's learning in the Chinese context. Their items captured Chinese parents' involvement by focusing on three learning‐related activities: arithmetic and mathematics, English alphabet, and Chinese characters. We included this three‐item scale with our seven‐item measurement. Results showed high reliability for both our seven‐item scale (α = .92) and Ip et al.'s (2018) three‐item scale (α = .92). Furthermore, the results indicated that scores on our seven‐item scale were significantly correlated (*r* = 0.69, *p* < .001) with scores on Ip et al.'s (2018) scale. In addition, each item of our scale was positively and significantly correlated with the latter, with correlation coefficients ranging from 0.46 to 0.67 (*p*s < .001). Taken together, our seven‐item scale showed convergent validity in assessing parents' involvement in children's learning activities.

#### Parental burnout

We measured parental burnout using the 23‐item Chinese version (Chen et al., 2022) of the Parental Burnout Assessment developed by Roskam et al. (2018). The scale consists of four dimensions: exhaustion in one's parental role (EX), contrast with previous parental self (CO), feelings of being fed up (FU), and emotional distancing from one's children (ED). Representative items are “I feel completely run down by my role as a parent” (EX), “I tell myself that I'm no longer the parent I used to be” (CO), “I can't stand my role as father/mother any more” (FU), and “I do what I'm supposed to do for my child(ren), but nothing more” (ED). Participants indicated how often they experienced these feelings in the past 3 months on a seven‐point rating scale (0 = *Never*, 6 = *Everyday*, α = .95). We averaged items, and higher scores indicated higher levels of parental burnout.

#### Flourishing

We measured flourishing using the eight‐item Chinese version (M. Tang et al., 2021; X. Tang et al., 2014) of the Flourishing Scale developed by Diener et al. (2010). The items were slightly adapted to reflect the family situation (e.g., “My family relationships are supportive and rewarding”). To capture the variations caused by PI‐E, participants were first asked to reflect on to what extent they had been feeling that way since the last survey (i.e., in the past month) and then answered on a seven‐point scale (1 = *Strongly disagree*, 7 = *Strongly agree*, α = .86). We averaged scores of all items to indicate participants' flourishing experience at home, and higher scores indicated higher levels of flourishing.

#### Creative process engagement

We measured creative process engagement with an 11‐item scale that was originally developed in the Chinese context by Zhang and Bartol (2010). The scale has three dimensions: problem identification, information searching and encoding, and idea generation. Example items include “I spend considerable time trying to understand the nature of the problem” (problem identification), “I consult a wide variety of information” (information searching and encoding), and “I consider diverse sources of information in generating new ideas” (idea generation). Again, to capture the variations caused by PI‐E, participants were first asked to reflect on to what extent they had been demonstrating each of the above‐mentioned behaviors since the last survey (i.e., in the past month) and then answered on a five‐point scale (1 = *Never*, 5 = *Very frequently*, α = .83). We averaged all the items, and higher scores showed higher levels of creative process engagement at work.

#### Work engagement

We measured work engagement using the nine‐item Chinese version (Fong & Ng, 2012) of the Utrecht Work Engagement Scale developed by Schaufeli et al. (2006). The scale has three dimensions (vigor, dedication, and absorption), each of which is measured with three items. Example items are “At my work, I feel bursting with energy” (vigor), “I find the work that I do full of meaning and purpose” (dedication), and “Time flies when I am working” (absorption). Similarly, participants were first asked to reflect on to what extent they had been feeling that way since the last survey (i.e., in the past month) and then indicated on a seven‐point scale (1 = *Never*, 7 = *Always*, α = .92). We averaged all the items, and higher scores indicated higher levels of work engagement.

#### Control variables

We controlled for variables that empirically or theoretically affect dependent variables in the present study. We controlled for job autonomy and job demands, two job characteristics that have been found to influence employees' engagement at work in prior studies (Bakker & Sanz‐Vergel, 2013; Sia & Appu, 2015). We measured job autonomy using the three‐item scale developed by Hackman and Oldham (1975): “The job permits me to decide on my own how to go about doing the work,” “The job gives me a chance to use my personal initiative and judgment in carrying out the work,” and “The job gives me considerable opportunity for independence and freedom in how I do the work” (1 = *Very inaccurate*, 7 = *Very accurate*, α = .81). We measured job demands with the four‐item scale used by Xie et al. (2008). Sample items are “My job requires me to work very fast” and “My job leaves me with little time to get things done” (1 = *Very inaccurate*, 7 = *Very accurate*, α = .80).

To rule out the possibility that the favorable outcomes of PI‐E were results of positive interactions with their children rather than the involvement in children's learning activities, we also controlled for participants' involvement in positive parent–child interactions at home. Indeed, Lin et al. (2021) suggested that participating in positive family events was positively associated with leaders' family need satisfaction, which also promoted their engagement at work. As their measurement items were targeting general family events (e.g., “I engaged in interesting activities with my family”), in the current study we shifted the reference to focus on parent–child interactions. Specifically, we used three items to measure positive parent–child interactions, which are “I play edutainment games with my children (e.g., card games, block games, board games, etc.),” “I do sports activities with my children,” and “I work on an art project with my children (e.g., handmade picture books, collages etc.).” Participants indicated the frequency of each item on a seven‐point scale (1 = *Never*, 7 = *Almost every day*, α = .73).

Finally, we controlled for several demographic and family characteristics. Participants' age and gender were included because these characteristics were shown to influence employees' family and work lives (Y. Tang et al., 2017). We also included whether the participant was the child's primary carer as a control variable, considering that parents who assume the primary caregiver role need to be involved not only in children's education but also in other daily life events related to children. Participants indicated whether or not they were their child(ren)'s primary carer at home (0 = *No*, 1 = *Yes*). Given that parents are more likely to enjoy, and therefore gain more resources from, being involved in their children's education if their children perform well in learning, we controlled for children's learning performance. Participants reported how well they thought their children were doing in the main subjects on a five‐point scale (ranging from 1 = *Struggling a lot* to 5 = *Very comfortable*). In addition, we controlled for participants' socioeconomic status (SES) because individuals with higher SES are more likely to value and be involved in their children's education (Jeynes, 2002), and can devote more resources to better engage in their work (Rubenstein et al., 2022). We measured participants' SES using the MacArthur Scale of Subjective Social Status developed by Goodman et al. (2001). Participants were shown a picture of a ladder with 10 rungs and were asked to indicate which rung of the ladder represented their status in their society. In the image, the top of the ladder (10) represents individuals with the highest education, income, and occupation status in their community, while the bottom of the ladder (1) represents individuals with the lowest status.

## RESULTS

### Descriptive statistics and measurement models

Table 1 displays the means, standard deviations, and correlations amongst variables in the present study. We examined the distinctiveness among the key constructs by conducting a series of confirmatory factor analyses. Given the multidimensionality and a large number of items of several variables (i.e., parental burnout, creative process engagement, and work engagement), to achieve a more optimal variable‐to‐sample‐size ratio, we adopted the internal‐consistency approach to construct internally consistent parcels for each variable (Little et al., 2002). Depending on the given number of dimensions, we created four parcels for parental burnout and three parcels for creative process engagement or work engagement, each parcel being the mean of all items for a given dimension. The hypothesized five‐factor model (i.e., PI‐E, parental burnout, flourishing, creative process engagement, work engagement) provided an acceptable fit: χ^2^(265) = 513.68; comparative fit index (CFI) = .92; root‐mean‐square error of approximation (RMSEA) = .07; and standardized root‐mean‐square residual (SRMR) = .06. We further compared this five‐factor model to a series of four‐factor models in each of which all items of either two variables loaded onto the same factor, whereas other variables remained intact. As expected, the five‐factor model fit the data better than any four‐factor models (*Δ*χ^2^s(4) ranging from 53.94 to 671.55, *p*s < .001; see Appendix S1), supporting the validity of our measurement model.

**TABLE 1:** Descriptive statistics and correlations.

Variable	Mean	*SD*	1	2	3	4	5	6	7	8	9	10	11	12
1. PI‐E (T1)	14.87	0.83												
2. Work engagement (T2)	15.07	0.92	−0.27***											
3. Creative process engagement (T2)	13.88	0.44	−0.18**	−0.66***										
4. Flourishing (T2)	15.97	0.60	−0.27***	−0.64***	−0.56***									
5. Parental burnout (T1)	10.75	0.72	−0.13	−0.29***	−0.34***	−0.39***								
6. Gender^a^	10.63	0.49	−0.17*	−0.02	−0.10	−0.02	−0.08							
7. Age	33.07	4.44	−0.02	−0.02	−0.05	−0.07	−0.01	−0.08						
8. Socioeconomic status	16.21	1.17	−0.16*	−0.35***	−0.32***	−0.36***	−0.18**	−0.01	−0.17*					
9. Job autonomy	15.69	0.77	−0.27***	−0.62***	−0.46***	−0.56***	−0.32***	−0.00	−0.11	0.28***				
10. Job demands	14.50	1.10	−0.01	−0.01	−0.06	−0.05	−0.06	−0.14*	−0.10	0.10	−0.12			
11. Positive parent–child interactions	13.68	1.05	−0.53***	−0.23***	−0.17*	−0.11	−0.02	−0.02	−0.11	0.18**	−0.21**	−0.05		
12. Children's learning performance	13.64	0.74	−0.13	−0.31***	−0.28***	−0.38***	−0.35***	−0.02	−0.02	0.30***	−0.26***	−0.03	0.10	
13. Children's primary carer^b^	10.51	0.50	−0.23***	−0.05	−0.05	−0.17*	−0.01	−0.37***	−0.13	0.06	−0.12	−0.11	0.01	0.04

*Note*: *N* = 206.

Abbreviations: PI‐E, parental involvement in children's education; *SD*, standard deviation; T1, measured at Time 1; T2, measured at Time 2.

* *p* < .05;

** *p* < .01;

*** *p* < .001.

^a^ Gender: 0 = male, 1 = female.

^b^ Children's primary carer: 0 = no, 1 = yes.

### Test of hypotheses

**FIGURE 2:**
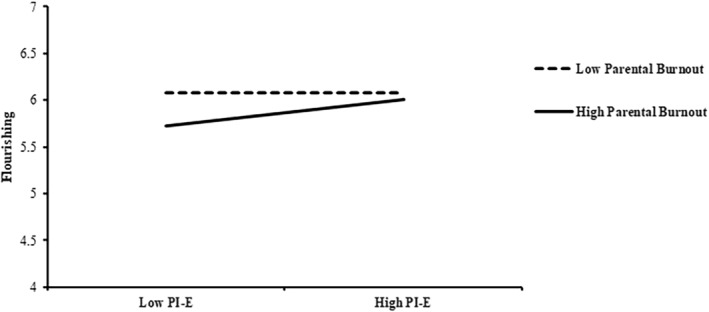
Interaction between parental involvement in children's education (PI‐E) and parental burnout on flourishing.

We tested our hypotheses using path analyses in Mplus and further constructed bias‐corrected confidence intervals with 5000 iterations to test hypotheses involving indirect effects using PROCESS in Mplus (Hayes, 2017; Stride et al., 2015). Table 2 presents all the results of the present analyses. In Hypothesis 1, we expected a positive association between PI‐E and flourishing. The result (Table 2, Model 1) showed that PI‐E was positively related to flourishing (*b* = .11, *SE* = .06, *p* = .07), but the effect was only marginal. However, this main effect was evidenced (Table 2, Model 2) by a significant interaction between PI‐E and parental burnout (*b* = .11, *SE* = .06, *p* = .049), such that a statistically significant positive relationship existed between PI‐E and flourishing when parental burnout was high (+1 *SD*, *b* = .17, *SE* = .07, *p* = .01) but not when it was low (−1 *SD*, *b* = .01, *SE* = .06, *p* = .93). The interaction pattern (Figure 2) was consistent with Hypothesis 2. Thus, the results lent support to Hypothesis 2 but not Hypothesis 1.

**TABLE 2:** Path analysis results.

Variables	Model 1: Mediation			Model 2: Moderation and moderated mediation		
	Flourishing	WE	CPE	Flourishing	WE	CPE
Control variables						
Age	−0.01 (0.01)	−0.01 (0.01)	−0.01 (0.01)	−0.01 (0.01)	−0.01 (0.01)	−0.01 (0.01)
Gender^a^	−0.08 (0.08)	0.01 (0.11)	0.13 (0.06) *	−0.07 (0.07)	0.01 (0.11)	0.13 (0.06) *
Socioeconomic status	0.08 (0.03)*	0.07 (0.04)	0.04 (0.03)	0.08 (0.03)**	0.07 (0.04)	0.04 (0.03)
Child learning performance	0.16 (0.05) ***	0.05 (0.06)	0.02 (0.04)	0.12 (0.05) *	0.05 (0.07)	0.02 (0.04)
Children's primary carer^b^	0.13 (0.07)	−0.11 (0.10)	−0.06 (0.06)	0.15 (0.08)	−0.11 (0.10)	−0.06 (0.06)
Positive parent–child interactions	−0.07 (0.04)	0.07 (0.05)	0.04 (0.04)	−0.05 (0.04)	0.07 (0.05)	0.04 (0.04)
Job autonomy	0.35 (0.05) ***	0.42 (0.11) ***	0.13 (0.05) **	0.32 (0.05) ***	0.42 (0.11) ***	0.13 (0.04) **
Job demands	0.04 (0.03)	0.00 (0.04)	0.03 (0.03)	0.05 (0.03)	0.00 (0.04)	0.03 (0.03)
Independent variables						
PI‐E	0.11 (0.06)^†^	−0.02 (0.08)	−0.04 (0.04)	0.00 (0.06)	0.02 (0.08)	−0.04 (0.05)
Flourishing		0.61 (0.12) ***	0.30 (0.06) ***		0.61 (0.12) ***	0.30 (0.06) ***
Parental burnout (PB)				−0.70 (0.27) **		
PI‐E × PB				0.11 (0.06) *		
*R* ^2^	0.430***	0.534***	0.395***	0.465***	0.534***	0.395***
Indirect effects [95% CI]		0.07 [−0.002, 0.15]	0.03 [−0.001, 0.08]			
Conditional indirect effects [95% CI]						
Parental burnout = +1 *SD*					0.10 [0.03, 0.22]	0.05 [0.01, 0.11]
Parental burnout = −1 *SD*					0.003 [−0.07, 0.11]	0.002 [−0.04, 0.04]
Difference					0.10 [0.01, 0.22]	0.05 [0.004, 0.12]

*Note*: *N* = 206. Unstandardized coefficients are reported, with standard errors in parentheses.

Abbreviations: CI, confidence interval; CPE, creative process engagement; PI‐E, parental involvement in children's education; WE, work engagement.

* *p* < .05;

** *p* < .01;

*** *p* < .001;

^†^ *p* < .10.

^a^ Gender: 0 = male, 1 = female.

^b^ Children's primary carer: 0 = no, 1 = yes.

Hypothesis 3 stated that positive associations existed between flourishing at home and both forms of engagement at work. The results (Table 2, Model 1) revealed that flourishing was indeed positively and significantly related to work engagement (*b* = .60, *SE* = .12, *p* < .001) or creative process engagement (*b* = .29, *SE* = .06, *p* < .001). Thus, Hypothesis 3 was supported.

Hypothesis 4 proposed that PI‐E would have two indirect effects on work engagement and creative process engagement through flourishing. The results (Table 2, Model 1) revealed that PI‐E had no significant indirect effect on work engagement (*b* = .07, 95% CI = [−.002, .15]) or creative process engagement (*b* = .03, 95% CI = [−.001, .08]) through flourishing. Thus, Hypothesis 4 was not supported.

**FIGURE 3:**
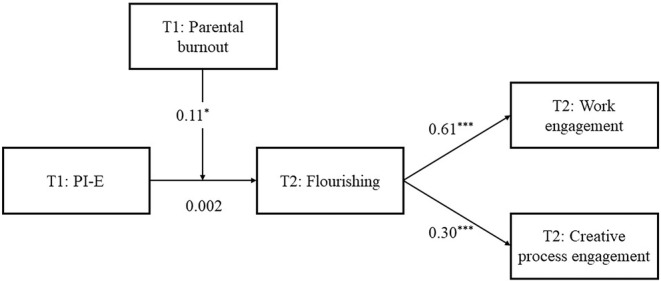
Path analysis results for the hypothesized model. Unstandardized coefficients are reported. For indirect and conditional indirect effects, see Table 2. PI‐E, parental involvement in children's education; T1, measured at Time 1; T2, measured at Time 2. *p < .05; ***p < .001.

Hypothesis 5 stated that the indirect effects of PI‐E on work engagement and creative process engagement through flourishing would be stronger when parental burnout was high as opposed to when it was low. Results (Table 2, Model 2) indicated that the indirect relationship between PI‐E and work engagement was significant and positive when parental burnout was high (*b* = .10, 95% CI = [.03, .22]) but not when it was low (*b* = .003, 95% CI = [−.07, .11]). Additionally, the result of pairwise contrast revealed that the difference of indirect effects between high and low levels of parental burnout was significant (*b* = .10, 95% CI = [.01, .22]). Similarly, the indirect relationship between PI‐E and creative process engagement was significant and positive for employees experiencing a high level of parental burnout (*b* = .05, 95% CI = [.01, .11]) but not for the counterparts experiencing a low level of parental burnout (*b* = .002, 95% CI = [−.04, .04]). Again, pairwise contrast revealed a significant difference of indirect effects between high and low levels of parental burnout (*b* = .05, 95% CI = [.004, .12]). Figure 3 shows the path analysis results for the full model. Taken together, Hypothesis 5 was supported.

## DISCUSSION

With the growing number of dual‐earner families, involvement in children's education at home for working parents has become increasingly common and necessary. Moreover, the need to investigate how PI‐E affects employees' experience in their family and work lives is also growing. The findings of the present study are counterintuitive: the positive effects of PI‐E on flourishing manifested only in parents who were already suffering from high levels of parental burnout, not in those with no or only mild symptoms of burnout. Contrary to our expectations, the hypothesized direct effect of PI‐E on flourishing was insignificant, and Hypotheses 1 and 4 are not supported.

According to the gain paradox principle of the COR theory, when individuals are in a resource‐loss state, the resource‐gain process would gain in value and become more salient (Hobfoll et al., 2018). Therefore, regarded as a parental resource‐loss state, employees with higher levels of parental burnout will experience more salient resource gain when they derive resources from their family. Similar findings have been observed in areas closely related to resources, such as organizational creativity. Y. Tang et al. (2017) found that individuals with high creative personalities, possessing abundant psychological and motivational resources for creativity, were less sensitive to the increase in family–work resource spillover. Conversely, people with low creative personalities were more sensitive to resource spillover from home to work, exhibiting creativity in the workplace. Besides, social support from friends (Madjar et al., 2002) and supervisor's developmental feedback (Zhou, 2003) contribute more to the creative performance of less‐creative employees, but less to that of highly creative employees.

Findings of the existing studies, together with the COR framework, might provide explanations for the insignificant main effect of PI‐E on flourishing. As mentioned earlier, PI‐E is also resource‐consuming in terms of time, energy, and money (Doepke & Zilibotti, 2019). For parents with low parental burnout, the perception of resource investment (e.g., energy, time) and resource gain (e.g., meaningfulness, fulfillment) of PI‐E may be in a balanced situation, resulting in the insignificant net effect of PI‐E on flourishing. Overall, our study provides supportive evidence for the gain paradox principle of the COR theory. These findings also advance our knowledge about when and how PI‐E could affect parents' well‐being and engagement in their work–family interface.

### Theoretical contributions

Our study makes several important contributions. First, we contribute to the eudaimonic well‐being and parenting literatures by indicating that PI‐E can be an important source of eudaimonic well‐being for burned‐out parents. Flourishing is a relatively new construct and most scholars in the parenting literature tend to focus on parents' emotions or hedonic well‐being (Nelson et al., 2014). A few studies have provided some insights into the relationship between parenting and some important components of eudaimonic well‐being, and these studies have primarily concentrated on general aspects of parenthood, such as childbirth (Brajša‐Žganec et al., 2022; Fave et al., 2013) or the time parents spend with children (Nelson et al., 2013). By demonstrating that PI‐E facilitates burned‐out parents' flourishing experience at home, our study suggests that PI‐E could be an important source of parents' eudaimonic well‐being.

Second, we contribute by identifying parental burnout as an important boundary condition that strengthens the positive influence of PI‐E on working parents' experiences at home and in the workplace. Most researchers tend to argue that parental burnout drives parents to withdraw from family events and interactions with children (Mikolajczak et al., 2019; Mikolajczak & Roskam, 2020). As a result, they are more likely to step into a state of chronic resource loss or even enter a downward spiral of resource loss. For individuals who have already suffered from resource loss, extant research suggests that the negative relationship between demands and outcomes will be worsened (Bakker et al., 2022; Valcour, 2007). Departing from existing research, we adopt a novel theoretical lens—more specifically, the gain paradox principle of COR (Hobfoll et al., 2018)—and reveal that parental burnout can function in a new pattern different from that identified in the literature. Specifically, our current study shows that although those burned‐out parents are seemingly under a disadvantageous resource‐loss condition, they are more likely to benefit from active engagement in their children's education. In this sense, our finding highlights the possibility that parents who are already in the resource‐loss condition can instead begin a resource‐gain process and even attain more resources than those who are in a favorable state of resource gain. By doing so, our work not only provides empirical evidence for COR's gain paradox principle (Hobfoll et al., 2018), but also answers the long‐standing call for shifting research attention to the moderating roles of personal or contextual resources in the relationships between work or family demands and the consequences (Voydanoff, 2002). Considering that it is only a preliminary finding, we encourage further exploration of the enrichment process amongst resource‐depleting individuals.

Finally, our study contributes to the PI‐E literature by extending its research scope. Extant research on PI‐E has largely been limited within the fields of developmental, educational, or school psychology, whose central interest is to investigate the effect of PI‐E on children's academic achievement (Fan & Chen, 2001; Hornby & Blackwell, 2018; Jeynes, 2007; Pomerantz et al., 2007). Given the increasing number of dual‐earner couples, handling both job demands and their children's educational needs is unavoidable for employees. However, to our best knowledge, few studies have investigated the potential beneficial effect of PI‐E for parents. By adopting a novel lens of the work–family interface, our study shows that PI‐E could help burned‐out parents maintain a better state, both at home and at work, even after considering their children's learning ability. This result indicates that parents could derive flourishing feelings from PI‐E, regardless of how their children behave. In other words, active engagement in children's learning process, in itself, can bring many positive results for working parents.

### Practical implications

First, our study provides practical implications for working parents who need to handle both work and home demands. Echoing Rudolph et al.'s (2021) notion that “the increased time spent with children provides unique opportunities for greater involvement of children in family activities and fosters improved emotional connections between parents and children that may improve long‐term family functioning” (p. 12), our work shows that PI‐E could enrich parents' flourishing experience and further improve their engagement at work. This finding not only encourages employees to actively engage in their children's education to attain flourishing feelings at home but also inspires organizations to initiate programs to encourage their workers to engage in PI‐E, which has beneficial spillover effects on employees' engagement at work.

Second, the present study inspires burned‐out parents to actively engage in children's learning activities. As Mikolajczak et al. (2021) point out, most intervention strategies that aim to mitigate the negative effects of parental burnout focus on societal (e.g., creating a society with fewer stressors on parents) and individual levels (e.g., changing parents' perceptions to decrease perceived stress). In the present study, our findings also found negative relationships between parental burnout and parents' socioeconomic status and their children's learning abilities (see Table 1), indicating potential social and personal stressors for parenting. However, our work suggests that, at the event levels, PI‐E could serve as a stress‐alleviating factor and help burned‐out parents gain valuable home resources. This finding encourages burned‐out parents to become bravely involved in high‐quality child‐related events (e.g., PI‐E) to gain resources and break the downward spiral of resource loss rather than simply escaping from their children.

### Limitations and future directions

Notwithstanding its contributions, the limitations of this study should be noted. First, we have argued that working parents could derive flourishing from PI‐E, which in turn could improve their work engagement and creative process engagement. Our study employed a two‐wave survey design with a 1‐month interval to measure core independent and dependent variables at two different time points. However, the causality of the present study may still not be confirmed. Also, the two‐wave design may not well capture the fluctuation of resources amongst working parents. Therefore, future studies could introduce it into longitudinal design or use the daily diary method to examine this causal relationship or uncover the subtle resource loss‐and‐gain process.

Second, in accordance with Erickson et al.'s (2010) operational definition of the school‐aged child stage, the present study targeted working parents with children in first to third grade in primary school (age 6–10 years). Existing studies suggested that appropriate PI‐E could benefit children's student outcomes throughout their schooling, including higher school years (Green et al., 2007). However, discussions about the effect of PI‐E for parents by age or grade differences were sparse. Therefore, whether our findings could shift for parents of older children may be questioned. As children move from the early school age into adolescence, they have a growing need for independence and an increased focus on peer relationships. Meanwhile, parents are less likely to be directly involved in their children's homework (Matza et al., 2001; Steinberg et al., 1992). From the COR theory perspective, the growing autonomy of children and the reduced involvement of parents may also result in parents' resource state of lacking family resources from their children, which is similar to those under the condition of parental burnout experiences. Thus, the effect of PI‐E may even be stronger for parents with children in the adolescent stage. Together with the limited implication of our findings because of the specific age group we focused on, studies with a broader range of children's ages would be preferred in the future.

Third, in the present study, we controlled for participants' positive parent–child interactions at home to rule out the possibility that the positive effect of PI‐E was just because working parents spent more time with their children. Although the significant effect of PI‐E remained after controlling positive parent–child interactions, we do not know exactly the different mechanisms between PI‐E and positive parent–child interactions. Therefore, further comparisons between PI‐E and other positive parent–child interactions may be preferred to investigate the different mechanisms and indicate the incremental validity of PI‐E on parents' work and family outcomes.

Fourth, although we regarded PI‐E as a resource‐gain process, our result indicated that parents with low parental burnout did not derive flourishing feelings from spending more time on PI‐E. Rothbard et al. (2021) indicated that enrichment and depletion both contribute to work–life balance and investigating how enrichment and depletion co‐occur to affect work–life balance is of significance. In accordance with this notion, we suggest that future research on PI‐E in the work–family interface should simultaneously take work–family enrichment and conflict into account. Further studies are necessary to fully understand how parental burnout influences the work–family enrichment or conflict processes.

Finally, we conducted our study in China, which has a Confucian culture where education is emphasized and valued (Kim et al., 2018). Previous surveys suggested that PI‐E has become an important component of family lives for parents with different cultural backgrounds (Herrold & O'Donnell, 2008; Williams et al., 2002). A 42‐country study also indicated the prevalence of parental burnout in different countries (Roskam et al., 2021). Given the widespread nature of PI‐E and parental burnout globally, coupled with the powerful impact of culture on both PI‐E (Corwyn & Bradley, 2008; Levine‐Rasky, 2009) and parental burnout (Roskam et al., 2021), replicating our findings in other cultural contexts is noteworthy. It is essential to explore how cultural variations may influence the relationships observed in our study, ensuring the generalizability and applicability of our findings across diverse cultural settings.

## CONCLUSION

Our study investigated the role of PI‐E in working parents' work–family interface. Parents who experienced higher levels of parental burnout could attain more flourishing feelings when they get more involved in their children's education. Moreover, for those suffering from parental burnout, the heightened flourishing experience they derived from PI‐E could further benefit their work engagement and creative process engagement.

## CONFLICT OF INTEREST STATEMENT

The authors declare there are no conflicts of interest.

## ETHICS STATEMENT

The current study was approved by the Institutional Review Board of the Institute of Psychology, Chinese Academy of Sciences (H20011).

## Supporting information


**Appendix S1.** Supplementary Information.

## Data Availability

The data that support the findings of this study are openly available in [Science Data Bank] at http://doi.org/10.57760/sciencedb.03112.
