# Supplementary material for: Recharging exhausted parents: How and when involvement in children's education increases working parents' flourishing at home and engagement at work
Source: Psych J. 2024 Apr 16;13(5):780–95. doi: 10.1002/pchj.753 (PMC11444731; doi:10.1002/pchj.753)
Supplement: Supplementary file 1 — Appendix S1. Supplementary Information. [file PCHJ-13-780-s001.docx]

**Recharging the exhausted parents: How and when involving in children’s education increases working parents’ flourishing at home and engagement at work**

## APPENDIX

Table A1. Results of confirmatory factor analyses

| Models | χ² | *df* | *Δχ^2^* | CFI | RMSEA | SRMR |
| --- | --- | --- | --- | --- | --- | --- |
| Five-factor model | 513.68^***^ | 265 |  | 0.92 | 0.07 | 0.06 |
| Four-factor model 1: PI-E and flourishing as a single model | 944.39^***^ | 269 | 430.71^***^ | 0.77 | 0.11 | 0.11 |
| Four-factor model 2: Flourishing and work engagement as a single model | 698.45^***^ | 269 | 184.76^***^ | 0.85 | 0.09 | 0.07 |
| Four-factor model 3: Work engagement and creative process engagement as a single model | 567.62^***^ | 269 | 53.94^***^ | 0.90 | 0.07 | 0.06 |
| Four-factor model 4: Creative process engagement and parental burnout as a single model | 760.42^***^ | 269 | 246.74^***^ | 0.83 | 0.09 | 0.12 |
| Four-factor model 5: PI-E and work engagement as a single model | 1095.47^***^ | 269 | 581.79^***^ | 0.72 | 0.12 | 0.15 |
| Four-factor model 6: PI-E and creative process engagement as a single model | 802.71^***^ | 269 | 289.03^***^ | 0.82 | 0.10 | 0.14 |
| Four-factor model 7: PI-E and parental burnout as a single model | 1185.23^***^ | 269 | 671.55^***^ | 0.69 | 0.13 | 0.14 |
| Four-factor model 8: Flourishing and creative process engagement as a single model | 616.24^***^ | 269 | 102.56^***^ | 0.88 | 0.08 | 0.07 |
| Four-factor model 9: Flourishing and parental burnout as a single model | 1046.89^***^ | 269 | 533.20^***^ | 0.73 | 0.12 | 0.10 |
| Four-factor model 10: Work engagement and parental burnout as a single model | 1121.40^***^ | 269 | 607.72^***^ | 0.71 | 0.12 | 0.11 |
| Single-factor model: All variables collapsed as a single construct | 1759.46^***^ | 275 | 1245.80^***^ | 0.49 | 0.16 | 0.15 |

*Note.* CFI = Comparative Fit Index; RMSEA = Root Mean Square Error of Approximation; SRMR = Standardized Root Mean Square Residual. *Δχ^2^* of each model was calculated and tested against the five-factor model.

^***^*p* < .001.
